# Diphtheria, Its Nature and Treatment

**Published:** 1861-03

**Authors:** C. W. Davis

**Affiliations:** Indianola, Iowa


### DIPHTHERIA,

### *Its Nature and Treatment.*

BY C. W. DAVIS, M. D., OF INDIANOLA, IOWA.

I have noticed several articles in the "Chicago Medical Examiner," other medical periodicals, and the usual Newspaper "Squibs," on the subject of *Diphtheria.* Having had a considerable amount of experience in the management of this disease with almost uniform success, I thought it would not be amiss, to give you ( and if you see proper ) to the readers of the "Examiner"—a statement as to the nature and treatment of this affection, so far as my humble opinion and experience go. In the spring of 1859, I was called to visit, in consultation, a case, the messenger said dying of croup. The patient was a boy between five and six years of age, had been sick some ten days, and at the time I saw him was laboring under all the phenomena of the last stage of croup. Frequent stridulous breathing, great anxiety, and restlessness—pulse over 140 with much thirst. I remarked to the attending physician, that I thought the boy would die in a short time, as I considered him laboring under infiltration into the cellular membrane of the Glottis—and that emetics would do no good. Tracheotomy would be the only resource, and that very doubtful. I was induced, however, to examine the fauces and throat, and there found several patches of ulceration—with a heavy black albuminous exudation—lining the entire throat, so far as I could discover. I immediately made a saturated solution of the sulphate of Zinc—adding some 30 drops of Elixir Vitriol to the ℥. Armed with a spoon (to compess the tongue,) and "swab" saturated with the solution, I prepared to swab out the throat. Introducing the spoon handle far enough back, the patient was forced to "gag." I immediately applied to the ulcerated portions of the throat, the swab, and was rewarded by bringing out with the instrument a large quantity of stiff, tenacious, and tough membrane, as well as a decided improvement in the patient's breathing. I will here say that I have used the solution of sulphate of Zinc in the various forms of Stomatitis, with marked benefit. Nitrate of Silver being less efficient, forming with albumen "the albu minate of Silver," and thereby becoming inert. I placed the patient on equal quantities of the Tinct's of Veratrum and Gelseminum, commencing with 4 drops, increasing 2 drops at a time, until the medicine produced its specific effect on the vascular and nervous system.

The bowels being in a constipated condition, and a want of tonicity of the mucous vessels, I gave alternately, powders composed of Quinine, Hyd. cum Creta, with a small quantity of Podophyllin until the bowels were freely evacuated, and then substituted for the Podophyllin, camphorated Dover's powder, made by adding the same quantity of powdered camphor as of opium and ipecac, in the old formula for making Dovers' powder. I ordered the throat to be swabbed out every two or three hours, with the Zinc solution, using at the same time cayenne peper liniment, composed of equal quantities of sweet oil, aqua ammonia, and turpentine with cayenne pepper in powder, to be applied freely to the external throat. On my next visit I found the patient decidedly better; pulse sixty-five per minute, respiration easy, ulcerations of throat more healthy, no albuminous exudation to be seen. By this course of treatment the patient recovered in a few days. There were several other members of the family laboring under Diphtheritic sore throat. The local applications of the Zinc solution and Liniment, soon cured them up. This form of stomatitis is attended with no particular febrile disturbance ,until the larynx and respiratory organs become affected. It is then, or rather it was then that the physician was first called. Should there be an impoverished condition of the fluids, general cachexy, the patient almost invariably dies of Oedema Glottidis—the tendency of Diphtheria if too long neglected. Since the Diptheritic "rage" I have made a great many useless visits, being called to see children with "cracked lips," "red gums," &c; their anxious parents supposing it to be Diphtheria. But this is all well enough, I have known this disease to run to a fatal issue, without the patient being bedfast more than two hours. The saturated solution of the *Super-Sulphate* of Zinc and cayenne pepper liniment, as topical applications with quinine and veratrum as a tonico-sedative will prove successful in a majority of cases, say eight out of ten.

